# Supplementary material for: Patterns of Intron Gain and Loss in Fungi
Source: PLoS Biol. 2004 Nov 30;2(12):e422. doi: 10.1371/journal.pbio.0020422 (PMC532390; doi:10.1371/journal.pbio.0020422)
Supplement: Table S1 — Also available at http://genes.mit.edu/NielsenEtAl/. (4.3 MB ZIP). [file pbio.0020422.st001.zip › NielsenEtAl/html/497.html]

AN1408.1.NCU02572.1.MG03616.1.FG09320.1


```
 CLUSTAL W (1.82) Multiple Sequence Alignments - Introns Inserted


Sequence 1: NCU02572.1	985 aa
Sequence 2: MG03616.1	793 aa
Sequence 3: FG09320.1	983 aa
Sequence 4: AN1408.1	985 aa
Alignment Length: 992 aa
Number Identitical Residues: 493 aa
Alignment Score (without introns) 26041


MG03616.1 	--------~-----~--------------------------------------------~
NCU02572.1	MDDDLYDE~FGNFI2GDAEASEEESEHG---VDAGNYVYDDYPEEAPEAPAQEMMEVDD~
FG09320.1 	-MDDLYDE2FGNFI~GEEVESEEGSEVG---VEAGDYVYDDEPDEAPGVTGQELMELDD1
AN1408.1  	-MDDLYDE2FGNYI~GEAAESDEEFQHEAAKPQAFDYDEAFGADELEEPAQQELMEVDE1
          	  ..  ..  ..   ..   :..  .  ::. .: .      ...    . ..  . .. 

MG03616.1 	------------------------------------------------------------
NCU02572.1	EGPSNAVVLHEDKQYYPTAAQVYGEGVEILVQEEDAQPLTQPIIAPVEQKKFSIEEADLP
FG09320.1 	-GPSNAIILHEDKQYYPTAEQVYGADVETRVEEEDAQPLSQPIIAPVEQKKFNIEEADLP
AN1408.1  	-GPSNAVILHEDKQYYPSAQQVYGEDVETLVQEEDAQPLSEPIIAPVTQKKFAIEESELP
          	 ..:.:    ....  .:: .  . . .   ....:.. :..  :.  ...   ..:. .

MG03616.1 	------------------------------------------------------------
NCU02572.1	PVYFDRTFMADLMNFPEQIRNVAFAGHLHHGKTAFMDMLVLETHDIADKLEKRTGKKRDE
FG09320.1 	PVFFDREFMTDLMNFPEQTRNVALAGHLHHGKTAFMDMLVLETHDITDRLERRVGKHRDE
AN1408.1  	PVFFSREFMTDLLNFPDQIRNIALVGHLHHGKTAFMDMLVMQTHDLAERLEKRTGRKRDE
          	.   .    :.  . ...  . :  .    ..::  .    .: . :.  .   .   ..

MG03616.1 	----------------------MSLVLESTKGKSHLFNMIDTPGHVNFVDEVASALRLVD
NCU02572.1	QLRYTDIHILERERGLSIKASPMSLVLQGTKGKSHLFNMIDTPGHVDFVDEVAASLRLVD
FG09320.1 	QLRYTDIHILERERGLSIKAAPMSLVLPSTKGKSHLVNLIDTPGHVNFVDEVAASFRLVD
AN1408.1  	QLRYTDVHHLERERGLSIKSAPMSFVLQSSKGKSHLFNIIDTPGHVNFVDEVAASSRLVD
          	.   :.    . . . : .::.**:** .:******.*:*******:******:: ****

MG03616.1 	GVCLVVDVLEGVQINTEQIIKHAVLEDIPMTLIVNKLDRLILELKLPPNDAYFKLKHAIE
NCU02572.1	GVCLVVDVVEGVQVNTEQIIKHAVLENIPLTLIVNKMDRLILELKLPPNDAYYKLKHVIE
FG09320.1 	GVCLVVDVVEGVQINTEQIIKHAVLEDIPLTLIINKMDRLILELKLPPKDAYFKLKHVVE
AN1408.1  	GVVLVVDVVEGVQANTEQIIRHAVLEDLPLTLVVNKMDRLILELKLPPNDAYFKLKHVIE
          	** *****:**** ******:*****::*:**::**:***********:***:****.:*

MG03616.1 	EVNTIITDTAVGQPGKAEKWRLSPEKGNVLFACSDMGWCFTLQSFSKFYADTYAPLDHTE
NCU02572.1	EVNTVIENT---IPGRGEERRLSPERGNVLFACTSMGWCFTLQSFAKMYSDTYGGVNSEE
FG09320.1 	EVNTIITNT---APTKAASKRISPEKGNVLFACTDMGWCFTLPSFAKMYTDTFGDINVDE
AN1408.1  	EVNTMIENV---MPGHGEKRRLSPEKGNVAFACASMKWCFTLQSFAKMYADTYPNIETAD
          	****:* :.    * :. . *:***:*** ***:.* ***** **:*:*:**:  ::  :

MG03616.1 	FARRLWGDIYFNPQKRSFTRKPVEEGAKRSFVKFVMEPIYKLFAHSISDSPEELKKVIGS
NCU02572.1	FARRLWGDIYFNPQKRSFTRKPIEEGAKRSFVNFIMEPIYKLYSHTISESPEELKGTLKK
FG09320.1 	FAKRLWGDIYYNPKKRNFSRKPIDERSSRSFVHFILEPIYKLFTHSISDSPEELKPVLAS
AN1408.1  	FAIRLWGDIFFNPRSRKFTRKGAEDNSKRTFVKFVLEPIYKLYSHTLSESPDDLKGTLAS
          	** ******::**:.*.*:**  :: :.*:**:*::******::*::*:**::** .: .

MG03616.1 	LGITLKPSQLRSDAQVLLRLVCQQFFGASSGFVDMVVAHIPSPVAAAEGRLQRYYTGPLD
NCU02572.1	LGIQLKPSQYKTDPKVLLKLVCEQFFGPSTGFVDMVCQHIPSPAEAAKQKLEQYYTGPLD
FG09320.1 	LGIELKPSQYKADAKVLLKLVCEQFFGPSTGFVDMIVKHIPSPIETAERLLERYYTGPLD
AN1408.1  	VGISLKPSQLRSDAKVLLDLVCEQFFGPAEGFVDMVLQHIPSPVEGAKRVLERYYTGPLD
          	:** ***** ::*.:*** ***:****.: *****:  *****   *:  *::*******

MG03616.1 	TEVAKSMASCNQDGPLVIQVTKLFSSPDAKTFHSFGRVMSGTATPGAEVRVLGEGYSVDD
NCU02572.1	SKVAESMLNCDQNGPLVLYVSKLFSAPDAKSFYSFGRVMSGIARPGTEVRVLGEGYSIDD
FG09320.1 	TKVAASMKTCDQDGPLVVHITKLFNTADAKSFHSFGRVLSGTVRPGMQVRVLGEGYSLDD
AN1408.1  	TKIAAAMANCDPDGPLVVHVTKLLANTDASRFHALGKILSGTARPGLQTRVLGEGYTPDD
          	:::* :* .*: :****: ::**:  .**. *:::*:::** . ** :.*******: **

MG03616.1 	EEDMAIARVSDIWISETKYNIPTDGVPAGNFVLLGGVDNSIVKSATIVDKTFENGEDAYI
NCU02572.1	EEDMAMGRISDVFIAETRYNIPTDGVPAGNWVLLGGVDNSIVKTATIVEKKFEDDEDAYI
FG09320.1 	EEDMAMANIAEVFIGETRYNIPTDGVPAGNLVLLSGVDNSIVKSATILPPKLEDDEDAYI
AN1408.1  	EEDMVNATISDTWIAETRYNIPTSGVTAGNLVLLGGVDNSIVKTATVVATKFEDNEEAHI
          	****. . ::: :*.**:*****.**.*** ***.********:**::  .:*:.*:*:*

MG03616.1 	FKPVTHFTQSVFKVAVEPINPSELPKMLDGIRKINKSYPLISTKVEESGEHVILGTGELY
NCU02572.1	FKPISHFTQSVVKVAVEPINPSELPKMLDGIRKINKSYPLITTKVEESGEHIIVGTGELY
FG09320.1 	FKPITHFTESVLKVAAEPINPSELPKMLDGLRRIQKSYPLIKTKVEESGEHVVLGTGELY
AN1408.1  	FRPIRHMTESVFKVAVEPVNPSELPKMLEGLRKVNKSYPLISTKVEESGEHIVLGTGELY
          	*:*: *:*:**.***.**:*********:*:*:::******.*********:::******

MG03616.1 	MDCVLHDLRRLYADMEIKVSDPVTRFCETVVEESATKCYAITPNKKNRITMVAEPLGDEI
NCU02572.1	MDCVLHDLRRLYADMEIRVSDPVVRFCETVQDMSATKCYAITPNKKNTITMVAEPLEDGI
FG09320.1 	MDCVLHDLRRLYADMDIKISDPVTRFCETVVETSATKCYAITPNKKNKITMVAEQLEKGI
AN1408.1  	MDCVLHDLRRLFSEMEIKVSDPVTRFCETVVETSAIMCYSITPNKLNKITMIAEPLDDGI
          	***********:::*:*::****.****** : **  **:***** * ***:** * . *

MG03616.1 	ARDIETGAVKIKDPVRKIAKFFQEKHEWDLLAARSIWAFGPEENGPNILQDDTLPTE~VD
NCU02572.1	AQDIESGAVRIRDPVRKIAKFFEEKYGWDLLAARSIWAFGPDEMGPNILQDDTLPSE0VD
FG09320.1 	SNDIESGAVNIRDPIRKTAKFFEEKHGWDKLAARSIWAFGPEETGPNILQDDTLPTE0VD
AN1408.1  	AEDIETGKVSIKDPIRKVARFFEEKYDWDKLAARSIWAFGPDELGPNILQDDTLPSQ~VD
          	:.***:* * *:**:** *:**:**: ** ***********:* ***********:: **

MG03616.1 	KKLLNTVKESIRQGFSWATREGPLCEEP1IRNTKFKIMDAALSQEAIYRGGGQIIPTSRR
NCU02572.1	KKRLNTVKDFIRQGFNWAVREGPLCEEP1IRNTKFRLIDVSLAQEAIFRGGGQIIPTSRR
FG09320.1 	KKTLNAVRESIRQGFSWATREGPLCEEP1IRNTKFKVTDVLLANEAIFRGGGQIIPTSRR
AN1408.1  	KKLLGSVRDSITQGFSWGTREGPLCEEP1IRNAKFRLTDVSLADQAIYRGGGQIIPTARR
          	** *.:*:: * ***.*..********* ***:**:: *. *:::**:*********:**

MG03616.1 	ACYSSFLMASPRLMEPTYAVSMIGSQDSVAQLYNTLARRRGHVLSDGPIAGTPLYRVSGL
NCU02572.1	ACYSSFLMASPRLMEPMYSVSMTGPQDAVSTVYNILARRRGHVLSDGPIAGTPLYRVNGL
FG09320.1 	ACYSSFLMASPRLMEPVYSVSVTGPEESYMEVYNVLSRRRGHVLSDGPVAGTPLYRVNGL
AN1408.1  	AVYSSFLMASPRLMEPLYSCSMTGPADAVASVYTVLSRRRGHVLSDGPIAGTPLYSVRGL
          	* ************** *: *: *. ::   :*. *:***********:****** * **

MG03616.1 	LPVIDSFGFETDLRIGSAGSATVSLVFDRWSIVPGDPLDREVVLRPLLPASAQATARDFV
NCU02572.1	LPVIDSFGFETDLRINTPGQAMVSLVFDRWNIVPGDPLDKEIVLRPLQMANAQATARDFV
FG09320.1 	LPVIDSFGFETDLRIKTQGSSMVSLVFDSWSIVPGDPLDREQIIRPLQPATAQATARDFV
AN1408.1  	IPVIDSFGFETDLRIHTQGQAAVSLVFDKWSVVPGDPLDRDVKLKPLEMAPAMATARDFV
          	:************** : *.: ****** *.:*******::  ::**  * * *******

MG03616.1 	LKTRRRKGLSEDVSVAKFLEPEFHSELISSGILGESM
NCU02572.1	LKTRRRKGLSEDVSVAKFLEPEFYQSLIESGTLGEA-
FG09320.1 	LKTRKRKGLSEDVSVKTFLEPEFYQSLMESGMLGEI-
AN1408.1  	LKTRRRKGLAEDVTVSKFLEPELWKGLKESGVLDS--
          	****:****:***:* .*****: . * .** *..
```
